# Reliability and validity of the Persian version of the ACE tool: assessing medical trainees’ competency in evidence-based medicine

**DOI:** 10.1186/s12909-022-03540-2

**Published:** 2022-06-17

**Authors:** Mohammad Amin Habibi, Mitra Amini, Maral Ostovarfar, Jeyran Ostovarfar, Mahsa Moosavi, Mohammad Hasan Keshavarzi

**Affiliations:** 1 Department of Radiology, Shiraz Medical School, Shiraz University of Medical Sciences, Shiraz, Iran; 2 Clinical Education Research Center, Shiraz University of Medical Sciences, Shiraz, Iran; 3 Department of Health Promotion, Shiraz University of Medical Sciences School of Health and Nutrition, Shiraz, Iran

**Keywords:** Medical student, Evidence-based medicine, Reliability and Validity

## Abstract

**Background:**

Evidence-based medicine (EBM) allows users to integrate evidence into decision-making alongside clinical expertise and patient values. This study aimed to evaluate the reliability and validity of the Persian version of the Assessing Competency in EBM (ACE) tool across knowledge, skills, and attitude.

**Methods:**

This cross-sectional study was performed on medical residents (first-year residents and junior residents) of Shiraz University of Medical Sciences in 2019. The study instrument was the ACE tool which consists of 15 two-choice questions (yes–no) and each of these questions measures one of four steps in evidence-based medicine (1- asking the answerable question, 2- searching the literature, 3- critical appraisal, and 4- applying the evidence to scenario). This tool was translated into Persian according to international standards. To ensure that the original and translated ACE questionnaire can be matched accurately and conceptuality, content validity index (CVI) and content validity ratio (CVR) were determined. Cronbach’s alpha was applied to determine the internal consistency for each scale and Confirmatory factor analysis (CFA) was used to survey the factor structure validity.

**Results:**

One hundred sixty-three questionnaires were studied, selecting 59 first-year medical residents and 104 s-year medical residents. The results showed that using the Persian translation of the ACE tools, the content validity index (CVI) values were equal to or above 0.8 for all items. The content validity ratio (CVR) value was 0.90 for the total scale. The indicators of the confirmatory factor analysis (CFA) for the ACE tool revealed that this model had an acceptable fit. Cronbach’s alpha for the overall score was 0.79.

**Conclusion:**

The Persian translated version of the ACE tool is a valid and reliable instrument for assessing medical trainees’ competency in EBM.

## Background

Evidence-based medicine (EBM) is a medical practice approach designed to optimize decision-making by focusing on evidence from high-quality clinical trials instead of more traditional sources of knowledge, like experts’ opinions, understanding of pathophysiology, or academic authority [1, 2]. EBM collects experience and proficiency from various disciplines, namely clinical epidemiology, information literacy, biostatistics, and knowledge management [3]. Many studies showed that practicing EBM is suitable for physicians to be experts, which contains a five-step process: “(i) constructing an answer to a clinical scenario question, (ii) systematic retrieving the best available evidence, (iii) “Critical appraisal of evidence for validity, clinical relevance, and application; (IV) use of results; and (v) performance evaluation” [3–5]. Given the growing importance of EBM, teaching EBM and training physicians who have the competence and skills to design and deal with a clinical question is necessary and a need [6].

In many universities and institutions operator of medical education, the EBM basics are part of the curriculum [7, 8]. However, the critical thing about different educational programs designed to teach EBM is that implementing these programs in various universities does not follow the same standard [9–11]. For example, in an institution, evidence-based medical education occurs in the first year of teaching [12]. Evidence-based medical education has been postponed for the last years [8]. Regarding the methods of evidence-based medical education, so far, a variety of ways of online teaching have been used until the training on patients’ beds [13].

Following the design and implementation of different educational programs and methods used for evidence-based medical education, it is necessary to evaluate learners to receive feedback and evaluate educational programs and practices. So far, several tools have been designed and used to assess the performance of individuals in EBM.

A systematic study in 2006 found that there were 104 unique tools for evaluating EBM, of which validity was established for only 53% [14]. In general, few tools measure or psychometrically assess all aspects of EBM, including knowledge, attitude, and skills [4]. The Berlin Questionnaire [15] and the Fresno Test [16] are the only two tools developed to date that assesses knowledge and skills across 3 of the 5 EBM steps.

In 2014, Dragan Ilic et al. Published a tool for the first time called assessing medical trainees’ competency in EBM, in which individuals’ skills are measured in four steps [4]. One of the strengths of this tool is assessing people’s ability to learn in-depth, which distinguishes it from similar tools due to the importance of evaluating people’s skills in EBM and the comprehensiveness of this questionnaire which has not been translated into Persian. It is a fact that among the educational groups, medical residents have more responsibility toward patients in our country than others; therefore, the importance of EBM for this group is twofold, and the introduction of a valid questionnaire can be a great help in this regard. This questionnaire also seems applicable in all medical schools and medical education centers. So we decided to translate this questionnaire into Persian for the first time in Iran and examine its validity and reliability among Shiraz University of Medical Sciences residents.

## Methods

### Study design and participants

This cross-sectional study was performed on 163 first and second-year medical residents of Shiraz University of Medical Sciences in 2019. Because of the questions’ duality and the statistical analyses, the required number of samples was estimated between 100 and 200 [17]. Given that the first and second-year medical residents are both junior students and do not differ much in terms of medical experience, 70 first-year medical residents and 110 s-year medical residents were selected. After collecting the questionnaires, 11 cases from the first-year medical residents and six from the second-year medical residents were excluded from the study due to incomplete questionnaires. Finally, 163 questionnaires were surveyed. Before starting the study, ethical clearance was obtained from the Shiraz University of Medical Sciences. Before initiating research activities, informed consent from participants was obtained. The confidentiality and anonymity of the data were guaranteed. We also informed the participants of their right to refuse to participate for any reason without penalty. Participants completed the ACE tool in person for over 60 min.

### ACE tool

The assessing competency in EBM (ACE) tool provides users with a brief patient scenario from which a clinical question is derived. Users are then presented with a search strategy (designed to identify a controlled randomized trial) and a hypothetical article extract [4]. The ACE tool consists of 15 two-choice questions (yes–no) that contain four dimensions:1- Items 1 and 2 were asking the answerable question, 2- items 3 and 4 were searching the literature, 3- items 5–11 were critical appraisal, and 4- items 12–15 were applying the evidence to scenario) [4]. Correct answers for the ACE tool questions had one point, and incorrect answers had a score of zero, with a maximum score of 15 and a minimum score of zero.

### Persian translation of the ACE tool

The ACE tool was translated according to the four sequential stages of translation and back translation as recommended by Chen et al. [18]. The instructions emphasized conceptual rather than verbal accuracy and used an acceptable linguistic approach for most Persian-speaking participants.

After obtaining permission from the authors of this questionnaire, the original version of the questionnaire was first translated into Persian independently by two people with Persian mother tongue and fluent in English. One of the translators was fluent in EBM, and the other translator was unfamiliar with these topics and the subject of the questionnaire. Agreements on translation differences were reached through dialogue between the two translators, and the final version of the questionnaire was prepared as a translation. Then, to ensure validity, the Persian version of the questionnaire was translated into English by two different translators who speak native English. One of them was familiar with EBM, and the other was not. The whole process of translation and back translation and coordination between translators was performed under the supervision of the study researcher.

After preparing the final version, a Persian questionnaire was given to some medical students fluent in EBM and unfamiliar with EBM to assess the face validity of the questionnaire. After the students completed the questionnaire, they shared their understanding of the questions through a conversation with the researcher. They also commented on the time to complete the questionnaire and its comprehensiveness. These steps’ results were reviewed in a meeting with researchers and included in the final version of the questionnaire with translators’ opinions.

### Statistical analysis

To specify the face validity of the Persian version of the ACE questionnaire, the questions were investigated in terms of their writing style, vividness, and fluency. So to ensure that the original and translated ACE questionnaire can be matched accurately and conceptuality, content validity index (CVI) and content validity ratio (CVR) were used. And also, they were equivalent to the original English ACE questionnaire. Hence to take content validity, 15 experts were hired to respond to each question of the questionnaire based on Lawshe to confirm the essentiality of the questions [19].

For this purpose, firstly, the questionnaires were distributed among the 15 faculty members. They were asked to assert their views about the necessity and appropriateness of each following question on the Likert scale (it is a necessary, a useful but not necessary, or not necessary). The CVI for each question was calculated using the formula: Total agreed points for each question/total number of participants. And for CVR, the formula was CVR = (Ne – N/2)/(N/2), where Ne was the number of agreed points for “essential” and N was the total number of participants [20].

All analyses were implemented in SPSS version 23 software (IBM Corp., Armonk, NY, USA) and AMOS version 23 software. The significance level of tests was considered equal and less than 0.05.

Cronbach’s alpha was applied to determine the reliability of internal consistency for each of those domains of the ACE tool and the total questions of the questionnaire. So, if the value of Cronbach’s alpha was equal to or greater than 0.70, it means that the reliability of each subscale was approved [21].

Confirmatory factor analysis (CFA) was used to explore the factor structure validity. Moreover, some criteria were implemented to determine the goodness of fit of the model, such as Chi-square statistics, root mean square error of approximation (RMSEA), comparative fit index (CFI), Tuker-Lewise index (TLI), Incremental Fit Index (IFI), Goodness of Fit Index (GFI), Comparative Fit Index (CFI), Normed Fit Index (NFI) and Adjusted Goodness of Fit (AGFI).

## Results

In total, 163 questionnaires were studied, selected from 59 first-year medical residents and 104 s-year medical residents. The results showed that the original version of the ACE tool could be applied to the Persian translation of the scale.

### Validity

The content validity index (CVI) values were equal to or above 0.8 for all items, and the content validity ratio (CVR) value was 0.90 for the total scale.

To assess the fitness of the final model of the ACE tool, confirmatory factor analysis was performed. The data were tested through CFA. Findings from the implementation of confirmatory factor analysis through eight evaluation criteria, including the value of the chi-square index, normed c2 measure index (the chi-square ratio of the degree of freedom), Goodness of Fit Index (GFI), Adjusted Goodness of Fit Index (AGFI), Normed Fit Index (NFI), Comparative Fit Index (CFI), Incremental Fit Index (IFI), Tucker-Lewis Index (TLI), and Root Mean Squared Error of Approximation (RMSEA), have been shown in Table 1.

**Table 1:** **The** indicators of fitness of the factor analysis of the ACE tool

Structure fitness indicators	**χ**^**2**^	***df***	**χ**^**2**^ ***df***	**GFI**	**AGFI**	**IFI**	**TLI**	**CFI**	**NFI**	**RMSEA**
Four-dimensional structure	162.098	84	1.930	0.875	0.822	0.910	0.884	0.907	0.826	0.074

**Fig. 1:**
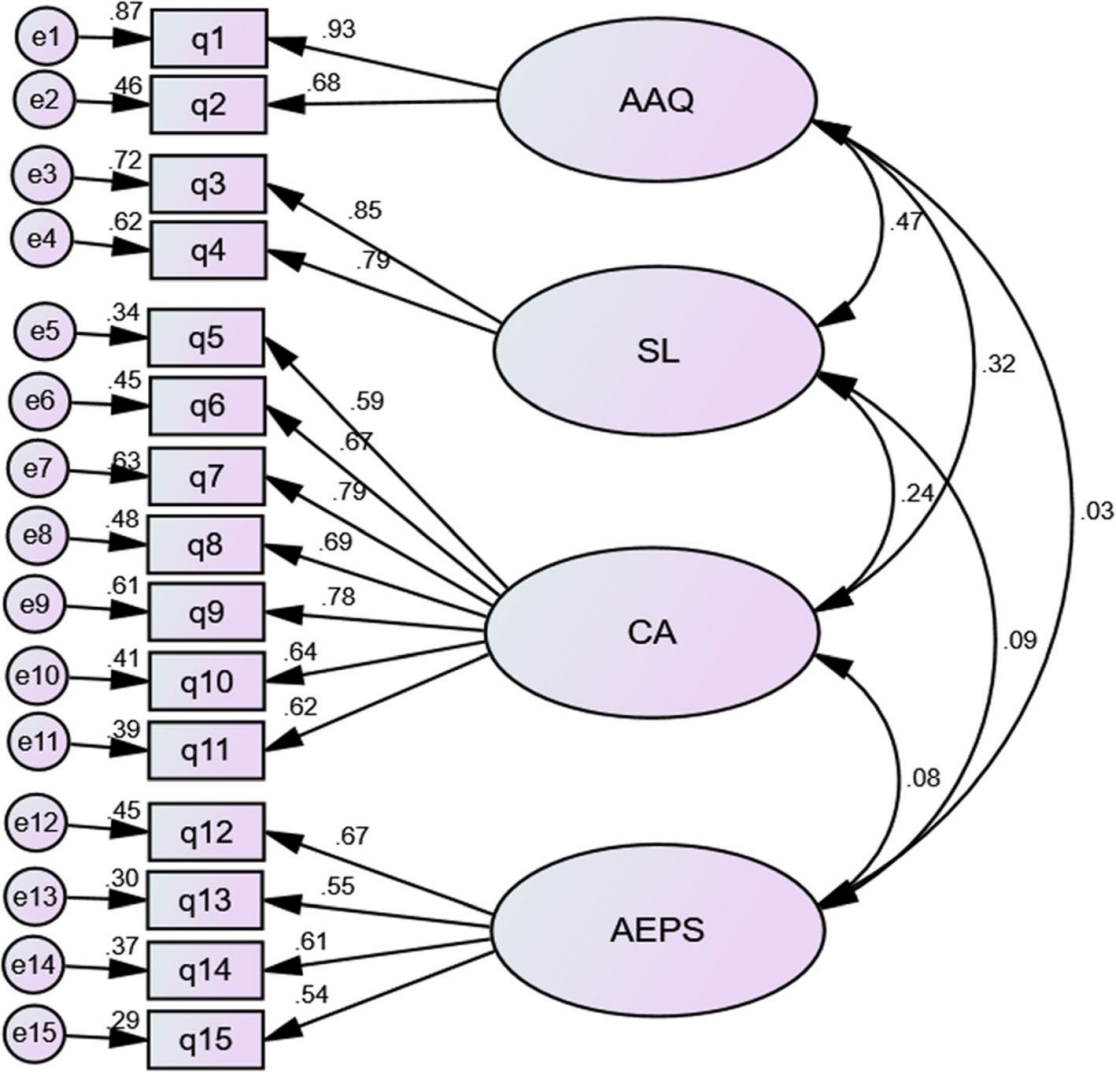
Factor structure of the ACE tool

The factor structure of the ACE tool in the present study has been presented in Fig. 1. Accordingly, all items had moderate to high factor loads (*p* < 0.001). Moreover, the indicators of the confirmatory factor analysis model of the ACE tool revealed that the indicators’ measures were close to the fitness criteria and that the CFA model had an acceptable fit.

### Reliability

Cronbach’s alpha was used to evaluate the ACE tool’s reliability, and the results have been reported in Table 2. Accordingly, the ACE tool had good reliability.

**Table 2:** The reliability coefficients of the ACE tool

Scale	Domains	Cronbach’s α	
ACE tool	Asking the answerable question	0.78	0.79
	Searching the literature	0.80	
	Critical appraisal	0.86	
	Applying the evidence to the patient scenario	0.70	

## Discussion

This study aimed to investigate whether the 4‐factor model from the original version of the ACE tool could be applied to the Persian translation of the scale. Because of this, the original version of the ACE tool was translated into the Persian language to identify its validity and reliability among first and second-year medical residents at Shiraz University of Medical Sciences. As a new tool for measuring EBM skills, the skill assessment tool in evidence-based medicine is different from other similar cases because, in addition to emphasizing and measuring in-depth learning, it measures four steps of evidence-based medical skills in a real patient scenario [4].

Internal consistency was determined to assess the reliability of the questionnaire. Internal consistency describes how many of the items of an instrument have the same concept or construct. Therefore it is connected to the inter-relatedness of the things within the test [22]. Using this approach, a Cronbach’s alpha coefficient of > 0.7 represents the instrument’s acceptable reliability [23]. In the current study, the reliability coefficient for the ACE tool was 0.79 and for Domains was 0.7 to 0.86. According to Bujang [23], these values indicated the acceptable reliability of the Persian version and are close to the results of a study by Dragan Ilic et al. [4].

The CVR was used to determine how experts agreed on the ACE tool questions translated into Persian. A CVR and CVI score of 0.80 indicates good content validity [24]. In the Persian ACE tool, the CVR value was 0.90 for the full scale, and CVI values were equal to or above 0.8 for all items. This study’s desirability of CVR and CVI values revealed that the Persian version of the ACE tool followed a proper and logical trend.

The fitness indicators of the confirmatory factor analysis model presented in Table 2 showed that ACE tool components of the Persian version had desirable conditions. The researcher used × 2 /df, GFI, and RMSEA among absolute fit indices, TLI, NFI, and CFI among other comparative fit indices. The results of these tests are × 2 / df ≤ 3, CFI and IFI > 0.9, TLI and NFI values are close to 0.9, and RMSEA < 0.08 indicates an acceptable fit [25]. Given the goodness-of-fit statistics values, the 4-subscales model fits the sample data, although the two indices (TLI and NFI) are a little below the threshold. Additional studies are necessary to adapt the 4-subscales model of ACE Tool for Iranian medical students. Furthermore, an inspection of the correlation between the loading estimates and the subscales in the path diagram shows the data fit the 4-factor model.

One of the limitations of this study was the lack of sufficient opportunities for medical residents to complete the questionnaire. The time required to complete the questionnaire was 60 min, and the questionnaires were conducted in the training centers. Second, this study was performed only in one institution (Shiraz University of Medical Sciences). Therefore, the generalizability of this study is limited. Also, assessing the competency of medical trainees in EBM is a new tool, so it is necessary to conduct similar studies in Iran and other countries to compare the validity and reliability of this study.

## Conclusion

The Persian translated version of the ACE tool is a valid and reliable instrument for assessing medical trainees’ competency in EBM that sets resident students’ skills in 4 steps. As Dragan Ilic et al. Noted in their study, implementing this tool is simple [4]. Of course, it is suggested that this study be performed on medical students of other medical universities in the country.

## Data Availability

The datasets used and analyzed during the current study are available from the corresponding author on request. The data are not publicly available due to privacy or ethical restrictions.

## Abbreviations

EBM: Evidence based medicine
ACE: Assessing medical trainees’ Competency in Evidence-based medicine
CVR: Content Validity Ratio
CVI: Content Validity Index
CFA: Confirmatory factor analysis
GFI: Goodness of Fit Index
AGFI: Adjusted Goodness of Fit Index
NFI: Normed Fit Index
CFI: Comparative Fit Index
IFI: Incremental Fit Index
TLI: Tucker-Lewis Index
RMSEA: Root Mean Squared Error of Approximation
